# Molecular Farming for Immunization: Current Advances and Future Prospects in Plant-Produced Vaccines

**DOI:** 10.3390/vaccines13020191

**Published:** 2025-02-15

**Authors:** Dang-Khoa Vo, Kieu The Loan Trinh

**Affiliations:** 1 College of Pharmacy, Gachon University, 191 Hambakmoe-ro, Yeonsu-gu, Incheon 21936, Republic of Korea; 2 Bionano Applications Research Center, Gachon University, 1342 Seongnam-daero, Sujeong-gu, Seongnam-si 13120, Gyeonggi-do, Republic of Korea

**Keywords:** plant-based vaccines, immunization, molecular farming, personalized medicine, infectious diseases, cancer vaccines, recombinant proteins, biopharming, vaccine development

## Abstract

Using plants as bioreactors, molecular farming has emerged as a versatile and sustainable platform for producing recombinant vaccines, therapeutic proteins, industrial enzymes, and nutraceuticals. This innovative approach leverages the unique advantages of plants, including scalability, cost-effectiveness, and reduced risk of contamination with human pathogens. Recent advancements in gene editing, transient expression systems, and nanoparticle-based delivery technologies have significantly enhanced the efficiency and versatility of plant-based systems. Particularly in vaccine development, molecular farming has demonstrated its potential with notable successes such as Medicago’s Covifenz for COVID-19, illustrating the capacity of plant-based platforms to address global health emergencies rapidly. Furthermore, edible vaccines have opened new avenues in the delivery of vaccines, mainly in settings with low resources where the cold chain used for conventional logistics is a challenge. However, optimization of protein yield and stability, the complexity of purification processes, and regulatory hurdles are some of the challenges that still remain. This review discusses the current status of vaccine development using plant-based expression systems, operational mechanisms for plant expression platforms, major applications in the prevention of infectious diseases, and new developments, such as nanoparticle-mediated delivery and cancer vaccines. The discussion will also touch on ethical considerations, the regulatory framework, and future trends with respect to the transformative capacity of plant-derived vaccines in ensuring greater global accessibility and cost-effectiveness of the vaccination. This field holds great promise for the infectious disease area and, indeed, for applications in personalized medicine and biopharmaceuticals in the near future.

## 1. Introduction

Over the past century, many changes have been observed on the international health scene. With these changes comes innovation in vaccine technologies to try and match increasing complexity [1,2]. Some of the big challenges for infectious diseases like COVID-19, influenza, and malaria are the development of new pathogens and the resurgence of vaccine-preventable diseases [3,4]. All inactivated vaccines and live-attenuated vaccines represent traditional approaches for vaccine manufacturing that have so far been remarkably effective [5,6,7]. Their limitations are becoming progressively more apparent, particularly with regard to preparation against pandemics [8,9]. Factors such as high production costs, reliance on cold-chain transportation, and manufacturing constraints slow down the rapid and fair distribution of vaccines, especially in low- and middle-income countries (LMICs) [10,11]. During the COVID-19 pandemic, the disparities in vaccine accessibility were felt more by the marginalized communities, hence exposing inherent vulnerabilities of the global health framework [12,13]. These problems reveal a great need for developing new vaccine technologies that are affordable, highly scalable, and deployable in a very short time against new health threats [14,15]. Plant-based vaccine technologies, through molecular farming advances, represent one of the innovative platforms well placed to fill these massive gaps [16].

Molecular farming is the process of using plants that are typically used in agriculture to produce proteins or other metabolites including plasma proteins, enzymes, growth factors, and recombinant antibodies, that have been heralded to revolutionize the approach of vaccine development [17,18,19]. In contradistinction to conventional production platforms, namely mammalian cell culture and microbial fermentation systems, molecular farming exploits the natural biosynthetic capabilities of plants in producing high-quality antigens for immunizations [20,21]. This is achieved either through transient expression systems, through stable plant transformation, or by chloroplast engineering towards efficient production of target antigens [22,23,24,25]. Transient expression systems, rely on the bacterial pathogen *Agrobacterium tumefaciens* as a powerful tool to introduce viral vectors or, in more advanced applications, to deliver components of a deconstructed viral vector into a host plant, allowing the shortest production cycles, and, therefore, uniquely suit responses to pandemics or outbreaks [26,27]. Indeed, Medicago’s plant-derived COVID-19 vaccine, Covifenz, has become the first such vaccine to be approved by regulatory authorities in Canada, a major milestone in the field [28,29,30,31]. In addition to human health vaccine production, molecular farming has been widely conducted in the field of veterinary medicine to produce vaccines—again proving to be more versatile and scalable [32,33,34]. The concept of edible vaccines, in which the antigenic proteins are produced in edible plants like lettuce or tomatoes, adds even another dimension in that there would theoretically not be any need for cold-chain distribution systems, thereby making vaccination more accessible under resource-constrained settings [35,36,37].

Molecular farming makes plant-based vaccine systems very attractive, as they can surmount the limitations of the traditional platforms of vaccine production [38]. First, plants could be grown in scale at a much lower cost, with no prerequisites for expensive bioreactors and stringent conditions for growth [39]. This is particularly the case for low- and middle-income countries where resources, both financial and technical, are in short supply [40]. Second, plant-based systems are free from the risk of contamination with human pathogens, which is an issue to some degree with mammalian cell-based production [41,42]. Plants are inherently safe biofactories since they cannot harbor or propagate viruses or prions that can infect humans [43]. Plant cell cultures are a controlled and scalable method of recombinant protein production independent of soil cultivation, with year-round production possibilities [44]. The bioreactor-grown systems provide a sterile, defined environment for growth with full control over the conditions, maximizing protein yield. Perhaps most importantly, they can produce complicated proteins with precise post-translational modifications, crucial for therapeutic proteins and monoclonal antibodies. Advances in genetic tools such as CRISPR and metabolic engineering have increased productivity [45,46]. Plant cell cultures are highly suited to vaccine antigens and biopharmaceuticals and offer consistency, flexibility, and scalability and are, thus, the prime focus of molecular farming strategies to produce biopharmaceuticals [47,48]. Third, plants are scalable, allowing very rapid ramp-up to meet surges in demand—such as those occurring due to pandemics [49]. Lastly, plant-based vaccines offer added advantages in that their production is more sustainable, producing a lower carbon footprint compared to traditional systems [50,51]. All of these characteristics make molecular farming a very powerful tool to achieve global health equity by making vaccines inexpensive and widely available [52].

This review will attempt to give an in-depth overview of the state-of-the-art of plant-based vaccine development, pointing out the important milestones, breakthroughs, challenges, and future directions of the field. First, it discusses the historical development and use of molecular farming in vaccine development. It then goes on to describe the mechanism of action of plant-based vaccine production in a detailed manner, covering major expression systems, host plants, and downstream processing techniques. Reviewing in more detail, the following describes various applications of plant-based vaccines in infectious disease prevention, cancer immunotherapy, veterinary medicine, advantages offered by the plant-based approach against the conventional vaccine platforms, and challenges that need to be overcome for the full exploitation of their potential. This work will hence outline the possible areas of research and innovation, discussing several needs for integrated approaches that bring together molecular farming with new delivery systems and improved biotechnologies. With such a dealing of topics, the present review tends to underline the turning point role that plant-made vaccines might play in changing the current situation in vaccine production and distribution worldwide.

## 2. Historical Perspective on Plant-Based Vaccines

The idea of a biofactory in plants for producing therapeutic proteins emerged at the beginning of the 1990s, encouraged by great achievements in molecular biology and genetic engineering [53,54]. In the early stages, research in molecular farming focused on the production of simple recombinant proteins, such as antibodies and enzymes, produced mainly in tobacco and potato [55,56]. In 1990, a breakthrough in human growth hormone production in transgenic tobacco plants showed that plants could serve as effective, scalable production platforms for proteins [57]. Not long thereafter, the first plant-derived vaccine antigen was expressed: a surface protein of the hepatitis B virus (HBV), both in transgenic tobacco and potato plants [58,59,60,61]. This indeed opened up the avenue to start exploiting plants in vaccine development. Several earlier studies related to edible vaccines expressed vaccine antigens in consumable crops like tomatoes and bananas as one such methodology to be conducted with simplified vaccine administration in the most resource-poor setting [62,63,64]. These experiments being critical for proving a number of plants as production platforms of immunologically active antigens marked the start of molecular farming as one of the revolutionary biotechnological approaches.

As the field evolved, researchers worked towards the optimization of yield, functionality, and immunogenicity of plant-derived vaccines, resulting in key milestones shaping the trajectory of molecular farming. Among the milestones marking this evolution has been the development of transient expression systems, capable of producing recombinant proteins in plants much faster without stable genetic modification [65,66,67]. This technique is a potent way to introduce viral vectors into host plants, usually by employing vectors from plant viruses like tobacco mosaic virus (TMV) or by using the bacterial pathogen *A. tumefaciens* [68,69]. While Agrobacterium-mediated plant transformation has been used since the 1980s, its use in recombinant protein production was initially limited by the low expression levels of foreign proteins [70]. The breakthrough with agroinfiltration truly changed the landscape of plant-based protein production [71]. This concept of rapid scalability of plant-based vaccines was first demonstrated through the transient expression of a candidate vaccine against the Norwalk virus in tobacco plants in 2005 [64,72]. The other major milestone was the expression of complex multimeric antigens in plants such as virus-like particles (VLPs) [73,74]. Due to a close mimicry of the native virus structure, VLPs are highly immunogenic and very effective in vaccine applications [75,76]. Successful plant-derived influenza and rotavirus VLP vaccines have reached clinical trials, further validating this technology for human immunization [77,78].

Medicago and GlaxoSmithKline (GSK) collaborated to create Covifenz, a plant-derived COVID-19 vaccine, which uses VLPs as its platform [79]. *Nicotiana benthamiana* expresses the SARS-CoV-2 spike protein, which allows for structural mimicry of the virus without its genetic material [80]. In this approach, plant cells were genetically engineered to produce proteins that self-assemble into VLPs. VLPs are mixed with GSK’s exclusive adjuvant AS03 to increase immunogenicity, which stimulates T cell and antibody responses [81]. The two doses of the immunization are spaced three weeks apart [82]. Covifenz was made using molecular farming, a technique that allows for quick scalability, a crucial benefit in pandemic response [30]. Phase III trials confirmed its effectiveness, and preclinical and clinical trials showed a strong immune response and a good safety profile. In February 2022, Health Canada granted emergency use authorization for Covifenz [31]. However, broader international approval was hindered by regulatory delays, commercial challenges, and competition from mRNA and protein subunit vaccines. Despite its innovative plant-based approach, which offers scalable and flexible vaccine production, Medicago’s closure in 2022 and limited market uptake raised concerns about the commercial viability of plant-based vaccines [83]. While Covifenz marked a significant milestone in vaccine technology, its market limitations underscore the need for further advancements to integrate plant-based platforms into global immunization strategies.

Beyond human health, veterinary medicine has also benefited, with the successful development of vaccines against animal diseases such as Newcastle disease and porcine epidemic diarrhea virus [84,85,86]. These show the wide applicability of plant-based platforms that keep being developed through new genetic engineering, synthetic biology, and improved technologies of downstream processing. Collectively, these signify the remarkable advancements in the evolution of plant-based vaccines, which hold the promise to transform healthcare on a global level.

## 3. Mechanisms of Molecular Farming

### 3.1. Plant Expression Systems

Plant expression systems have been developed as the major tools in molecular farming for only a few approaches regarding the production of recombinant proteins, including vaccines [87]. The two prevailing methodologies hitherto being explored in this domain include transient expression and stable transformation [21,88,89]. Both possess certain peculiar advantages and applications in molecular farming.

During plant-based production of vaccines, viral vectors generally introduce target proteins into the plant cells. Agroinfiltration is one of the most applied techniques wherein the bacterium *A. tumefaciens* is utilized as a vehicle to deliver viral vector constructs encoding the desired protein due to its natural capacity to transfer DNA into plant cells [90,91,92]. Because it will express the protein of interest in days, this methodology offers the advantages of transient expression, and can, therefore, represent an efficient methodology towards fast vaccine production. Among the well-known transient expression systems, one is based on the vectors from the Potato Virus X [93]. PVX is a plant virus that replicates in a wide range of plant species, thus, being an ideal vector for the production of proteins [94]. The systemic expression of the target protein induced by this virus in infected plant tissues allows recombinant proteins to be easily harvested. These vectors are very helpful in large-scale production due to the less time-consuming process and low cost compared with traditional expression systems in mammalian or bacterial cells. Agroinfiltration and PVX-based vectors have contributed much to plant molecular farming, enabling scalable production of recombinant proteins, including vaccine candidates. In addition, both systems are currently under investigation for flexibility and efficiency, which will extend their wider applicability in biotechnological and pharmaceutical areas.

In the case of stable transformation, however, the gene of interest has to be integrated into the plant genome to produce transgenic plants that can express the required protein in subsequent generations [95]. This is achieved by nuclear or chloroplast transformation. Nuclear transformation inserts genes into the plant’s nuclear genome; this can take place with Agrobacterium-mediated transformation and biolistic (gene gun) methods [96,97]. Transgenic plants provide long-term production of proteins without repeated introduction of the gene. However, high-level expression and uniform yields are not always easy to achieve because of position effects and gene silencing [98,99]. Chloroplast transformation overcomes many of the limitations associated with nuclear transformation because integration of the gene into the chloroplast genome allows for higher levels of protein expression [100,101]. Chloroplasts can carry multiple copies of the gene of interest, and the lack of gene silencing enhances stability. Furthermore, the risk of transgene escape through pollen is minimized in chloroplast transformation because chloroplasts are maternally inherited in most plants [102]. By such means, antigens against a range of diseases such as cholera, tuberculosis, and anthrax have been fashioned into vaccine candidates [103]. Compared to transient systems, stable transformation is slower, but it comes into its own in long-term production and applications that require large-scale cultivation of plants. These plant expression systems now come together to give a strong molecular farming toolkit for the development of plant-based vaccines for diverse needs.

### 3.2. Plant Hosts in Molecular Farming

The choice of the plant host is one of the critical parameters affecting the efficiency, scalability, and economic viability of the process in recombinant protein production with molecular farming [104]. Examples of host plants include tobacco, lettuce, tomatoes, and maize, each having distinct advantages and certain limitations [105]. They are mainly chosen with considerations in their growth rate, yield of the target protein, capabilities in relation to post-translational protein modification, and appropriate application accordingly.

#### 3.2.1. Commonly Used Plants

Tobacco (*Nicotiana* species) is the most exploited plant for molecular farming, mainly because of its well-developed genetic transformation protocols and high biomass yield [106,107]. *N. benthamiana* has been the choice for transient expression systems since it supports high levels of recombinant protein production and is amenable to viral vector-based approaches [108]. In addition, tobacco plants are fast-growing and not part of the food chain for human consumption, thus, lessening the possibility of contamination of edible crops with recombinant proteins. On the other side, the use of tobacco in vaccine production calls for thorough purification since it contains alkaloids and other contaminants that may interfere with the final product [109].

Lettuce (*Lactuca sativa*) has been the focus of research as a host for edible vaccines [110]. Its leaves are edible in the raw state, thus, serving as a good candidate for the oral delivery of antigens with minimum processing [111]. Antigens against diseases such as hepatitis B and norovirus have been expressed in lettuce [112]. However, there is always a problem in obtaining consistent levels of protein expression in lettuce, and there is always a possibility of degradation of the antigens in the gut [113].

Tomatoes (*Solanum lycopersicum*) have also been explored for edible vaccine production [114]. Widespread cultivation, ease of transformation, and ability to accumulate high levels of recombinant proteins in the fruit make tomatoes convenient as hosts [115]. The antigens expressed in tomatoes are able to be stored in either a desiccated or powdered form for longer periods of time because of improved stability and utility [116]. The productivity of tomatoes is relatively low compared to tobacco, and fluctuating expression levels of transgene between batches of fruit limit large-scale industrial application [117,118].

Maize (*Zea mays*) is one of the most widely used cereal crops and has great potential for molecular farming, especially for stable transformation and large-scale production of therapeutic proteins and vaccines [119]. Maize seeds provide natural encapsulation, which protects the recombinant proteins from environmental degradation and gastrointestinal enzymes, thus, being suitable for oral delivery [120]. The scalability of maize cultivation and its compatibility with the existing agricultural infrastructure make it attractive for cost-effective production. However, the other major drawbacks include gene flow to wild relatives and food crops, besides the relatively slower transformation process [121].

#### 3.2.2. Comparative Analysis of Plant-Based Systems with Other Expression Platforms

Plant expression systems benefit and are limited compared to animal, fungal, and bacterial systems.

Compared to bacteria (e.g., *E. coli*), which are good at producing simple proteins, plants are better at producing complex proteins with proper folding and post-translational modifications, e.g., glycosylation [122]. Plants provide glycosylation patterns closer to human systems than yeast or fungi and are, therefore, better suited for biopharmaceuticals like antibodies. Plants generally take longer production times compared to microbial systems, which can be rapidly scaled up for the production of less complex proteins.

Fungal systems, for example, Pichia pastoris, combine high rates of growth with eukaryotic-like protein processing but yield hypermannosylated glycoproteins preferentially, which can cause immunogenicity in human beings [123]. Plants occupy this niche by providing a compromise between cost, scalability, and human-compatible glycosylation. Despite these benefits, plant systems are also characterized by low protein titers and longer expression times compared to microbial and fungal systems.

Animal systems, including mammalian cell cultures, are the preferred choice for high-value biopharmaceuticals due to the potential to offer human-like post-translational modifications [124]. These systems are expensive, require sophisticated bioreactor facilities, and are also prone to contamination with human pathogens. Plants offer the safer, less expensive option with reduced risk of contamination and the potential for open field or closed system scale-up.

## 4. Applications of Plant-Based Vaccines

### 4.1. Infectious Diseases

Plant-based vaccines have had quite a few successes in fighting infectious diseases, including COVID-19, influenza, hepatitis B, and malaria. During the COVID-19 pandemic, Covifenz used plants of *N. benthamiana* to produce VLPs with a striking similarity to the SARS-CoV-2 spike protein [125]. Similarly, plant-produced influenza vaccines have shown good immunogenicity [126]; hemagglutinin antigens and VLPs expressed in tobacco plants are now in advanced clinical trials [127].

Early hepatitis B research focused on the expression of the surface antigen (HBsAg) in transgenic plants such as tobacco and potatoes, showing great promise as an alternative to traditional vaccines [128,129]. In present-day research, plant-based research into hepatitis B vaccines has moved beyond the initial focus on HBsAg. In fact, several efforts are going in the direction of a greater emphasis on recombinant VLPs and other immunogenic proteins for higher vaccine efficacy [129,130].

**Figure 1:**
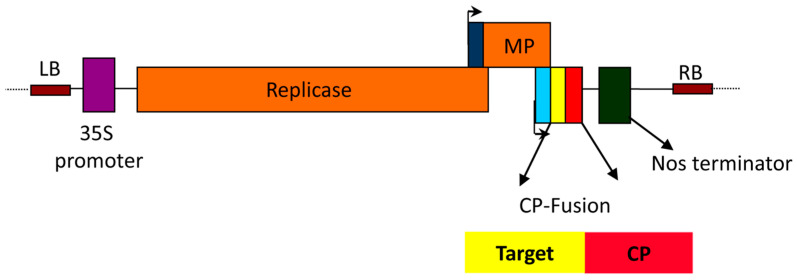
Schematic diagram of the ‘launch’ vector. Following agroinfiltration of plants, the sequence between the left border (LB) and the right border (RB) of the plasmid vector is transferred from Agrobacteria into plant cells where expression of the engineered TMV genome is driven by the Cauliflower mosaic virus (CaMV) 35S promoter. TMV replicase then drives the amplification of the primary transcript, and Pfs25-CP accumulation is then driven by the TMV CP subgenomic promoter (light blue box). The movement protein (MP) facilitates cell-to-cell transfer of viral sequences and is driven by the MP subgenomic promoter (dark blue box). “CP” refers to “coat protein”. Copyright Public Library of Science (2013) [28].

A promising malaria transmission-blocking vaccine (TBV) was developed using VLPs produced in N. benthamiana via a TMV-based ‘launch’ vector. The vaccine targets Plasmodium falciparum Pfs25, a key protein on gametes, zygotes, and ookinetes. Jones, R.M. et al. engineered hybrid VLPs (Pfs25-CP VLPs) by fusing Pfs25 to the Alfalfa mosaic virus coat protein (CP), achieving 20–30% Pfs25 incorporation on VLP surfaces. Mice immunized with Pfs25-CP VLPs plus Alhydrogel^®^ developed strong, sustained antibodies with complete transmission-blocking activity for six months. This study demonstrates the potential of plant-based VLP vaccines for malaria control [28] (Figure 1).

Vaccination against infectious diseases continues to be the central point of vaccine manufacturing, including vaccines produced in plants that are cost-effective, scalable, and safe alternatives to conventional vaccine systems [131]. Plants are biofactories where recombinant antigens are expressed in the form of VLPs, subunit vaccines, and monoclonal antibodies that confer protective immunity [42]. Some vaccines based on plants have shown encouraging preclinical and clinical outcomes, mainly for viral and bacterial pathogens. The advantages of plant-based vaccines include rapid production, potential for oral administration, and reduced chances of contamination with human or animal pathogens. The subsequent table gives an overview of significant examples of plant-based vaccine candidates (Table 1).

### 4.2. Edible Vaccines

**Figure 2:**
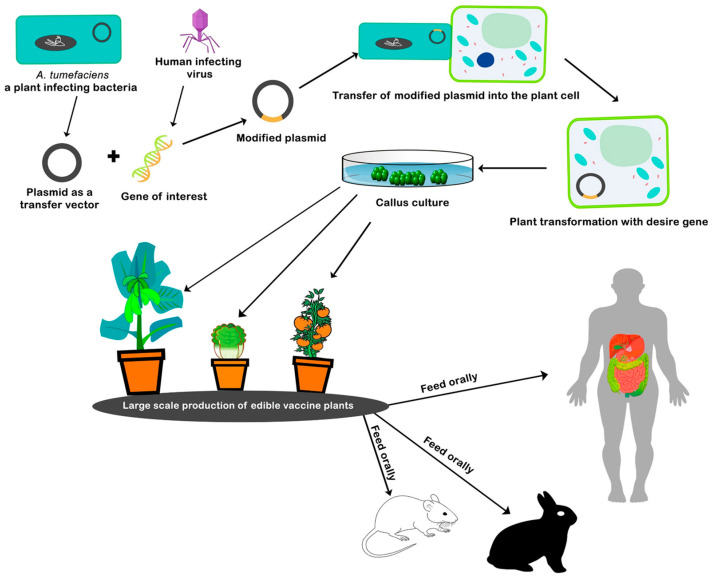
The procedure involved in the development of the edible vaccine. Copyright Elsevier (2020) [35].

Edible vaccines, produced using plant-based systems, represent a promising innovation for low-resource settings where the traditional infrastructure for vaccine production and distribution has been insufficient [139]. Plants like bananas, tomatoes, potatoes, and lettuce have been engineered to produce antigens from pathogens [35] (Figure 2).

Edible vaccines, however, have a number of disadvantages. One of the big challenges in using plants to produce vaccines is the inconsistent level of antigen expression, as variable growing conditions can affect protein yield [140]. Furthermore, it is difficult to deliver a precise dose of antigen because of variability in the size and composition of the edible plant material [141]. The stability of antigens during storage and transit, and their degradation in the human digestive system, are other important issues that need to be overcome. Other strategies being considered to improve efficacy and stability involve freeze-drying plant material or encapsulating antigens to protect them from being destroyed by the stomach’s digestive enzymes [142,143].

The success of edible vaccines depends on the delivery mechanism. Direct consumption of raw edible plants contributes to improved stability of the antigens, and the mechanism provides a controlled dosage in processed forms such as powders and capsules. Such mechanisms are very challenging for edible vaccines, but this is one clear area where efforts should focus on plant-based vaccine research related to diseases most affecting underserved populations.

### 4.3. Therapeutic Vaccines

Therapeutic plant vaccines utilize the immune system to cure disease either by stimulating immune responses against chronic infections like cancer and viral infections or by inducing immune tolerance against autoimmune diseases. In contrast to prophylactic vaccines, which immunize the immune system against infection, therapeutic vaccines introduce disease-specific antigens to the immune system to modulate immune function [144].

For cancer and chronic infections, plant-based vaccines that express tumor-associated antigens (TAAs) or pathogen proteins stimulate cytotoxic T lymphocytes (CTLs) to recognize and kill cancer cells [98,145,146]. Plant-derived vaccines, for instance, have been engineered to express antigens such as HER2, a protein overexpressed in breast cancer, or mucin-1, a glycoprotein frequently expressed in ovarian and breast cancer; such vaccines have shown potential in preclinical studies as immunotherapeutic agents for cancer treatment [147,148]. The work by McCormick et al. in their 2008 PNAS-published Phase I clinical trial of plant-produced therapeutic vaccines for non-Hodgkins lymphoma is another relevant instance [149]. This phase I clinical trial showed that plant-derived idiotype vaccines are feasible, safe, and immunogenic in patients with follicular B-cell non-Hodgkin’s lymphoma. Using a viral expression system of plants, tumor-derived personalized single-chain antibodies were prepared from each individual patient in a relatively short time, thus, making individualized immunotherapy possible. Vaccines administered either without or with the GM-CSF adjuvant were well tolerated with no serious adverse events. Resultant immune responses for over 70% of patients included antigen-specific responses induced in 47%. Of great importance, different glycosylation of plant-produced antigens neither impeded the immunogenicity nor impinged on safety. These plant-based idiotype vaccines are placed among the hopeful and feasible options for lymphoma immune therapy.

For autoimmune diseases such as type 1 diabetes and multiple sclerosis, therapeutic plant-based vaccines induce tolerance by expressing self-antigens in a regulated manner [150,151]. By this mechanism, the immune system is reeducated and autoimmune responses leading to destruction are abrogated. Oral administration is especially advantageous in these situations since plant vaccines can be targeted against the gut-associated lymphoid tissue (GALT) to activate regulatory T cells (Tregs), the cells responsible for immune tolerance [152,153]. This strategy has been explored in allergy and inflammatory bowel disease, in which plant vaccines can modulate mucosal immunity and abolish inflammation and hypersensitivity reactions. Outside of infectious and immune diseases, plant vaccines are being developed for neurodegenerative and chronic disease states such as Alzheimer’s and cardiovascular disease. Anti-amyloid-beta plaque vaccines have been developed in plants to elicit an immune response that would help eliminate plaques in Alzheimer’s patients [154]. Therapeutic vaccines against cholesterol metabolism are also being researched to avert atherosclerosis and cardiovascular disease risk [155].

The use of plant-based vaccine development is expanding into chronic infectious diseases such as HIV and tuberculosis [111,156]. Plant-derived HIV vaccines frequently contain envelope glycoproteins or conserved viral epitopes that generate neutralizing antibodies and CTL responses, with the goal of reducing viral load and preventing disease progression [157]. Similarly, plant-based tuberculosis vaccines produce important Mycobacterium tuberculosis antigens, such as Ag85B or ESAT-6, to boost Th1 immune responses [158].

**Figure 3:**
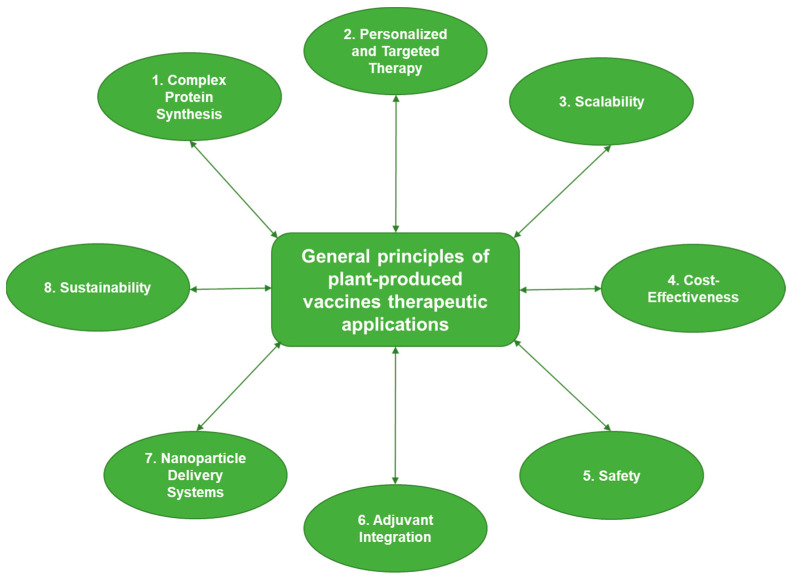
The general principles of plant-produced vaccine therapeutic applications.

Immunotherapies using plants for chronic diseases are still in their infancy; however, the versatility of plant systems in producing diverse therapeutic proteins does hold much hope for future advances. These developments have emphasized the great potential of plant-based vaccinations for therapeutic purposes and marked a new generation of immunotherapy that is inexpensive and scalable for wide application in the treatment of cancer and chronic diseases (Figure 3). This will open ways to produce, in plants, disease-specific antigens or modulatory proteins, for the generation of vaccines that will prevent infection, induce immune tolerance, or restore immune function in the case of chronic diseases.

### 4.4. Veterinary Applications

**Figure 4:**
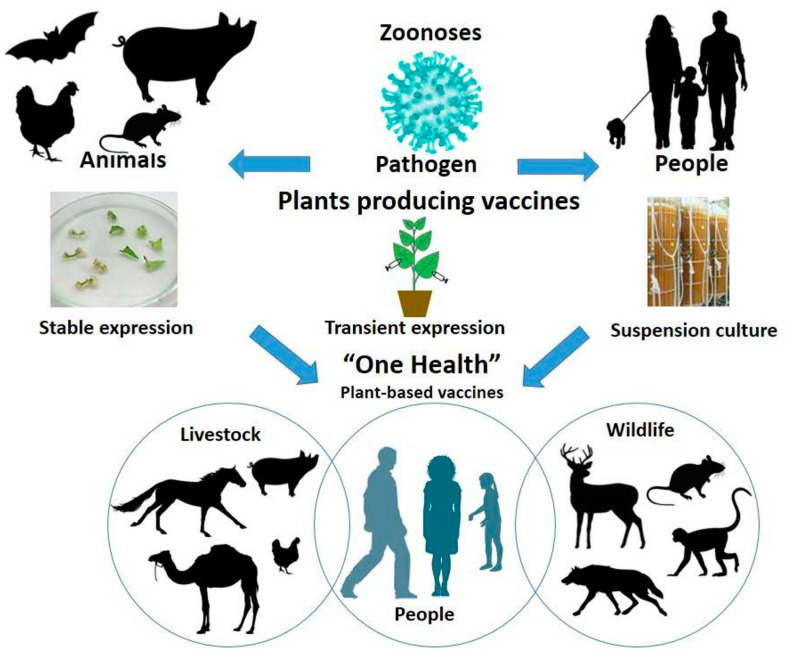
Schematic representation of the transmission of zoonotic diseases and the plant-based production technologies (stable, transient, and suspension cultures) for recombinant vaccine production. The figure contains the reprinted disposable bioreactor image from PROTALIX Biotherapeutics under the Copyright MDPI (2022) [160].

Plant-based vaccines have tremendous potential in veterinary medicine and for the prevention of zoonotic diseases in humans [159,160]. Such vaccines could be engineered against specific animal pathogens like viruses, bacteria, and parasites [161,162,163,164]. Vaccines could also be more effectively delivered using plants. Indeed, there are great successes reported for protection against avian influenza in poultry, swine fever in pigs, and rabies in cattle with the administration of plant-derived vaccines. Scientists using these plant expression systems, particularly the *N. benthamiana* or *N. tabacum*, have produced immunogenic proteins such as VLPs or recombinant antigens that elicit strong immune responses in animals [162] (Figure 4). Vaccines are scalable and inexpensive and can meet the increased demand for animal vaccines in developed and developing regions.

The “Global One Health” paradigm is a model of the interconnectedness of human, animal, and environmental health [165]. It puts the promotion of collaboration and interdisciplinarity into action to meet global health challenges such as animal-borne diseases, antimicrobial resistance, and emerging pandemics. This paradigm will be relevant to plant-based vaccines since it would be able to prevent diseases of humans and animals with sustainability and lower environmental impact. Table 2 provides a summary of notable examples of plant-based vaccines that have been developed and applied in veterinary medicine, highlighting their potential in preventing various animal diseases.

When such infection in an animal is controlled through a plant-derived vaccine, that would mean a reduction in zoonotic outbreaks, which may have devastating effects on human health. Examples of such plant-based vaccines target diseases like *E. coli* and Foot-and-Mouth Disease, FMD, show promise for stopping the livestock-to-human transmission of these pathogens, where this is often the case between free-roaming livestock and people living in very close proximity to the livestock herds [170,171]. Additional benefits of the new vaccines involve delivery as part of edible plants, such as fruits or feed; this mode of administration eases delivery by obviating injections, often a barrier when veterinary infrastructure is rudimentary or missing [172].

The continued development of plant-based vaccine technology has great potential to improve global livestock health and prevent zoonotic disease transmission; hence, an innovative solution to modern agricultural challenges.

## 5. Benefits of Plant-Based Vaccines

### 5.1. Cost-Effectiveness and Scalability

Economic feasibility and probable large-scale production, some of the most important advantages, make the plant-based vaccine very attractive in generating mass vaccines [173]. This will be considered with the traditional approach to vaccine production, which usually involves expensive bioreactors among other infrastructures. Plant expression systems take advantage of the plants to produce proteins relatively at a much lower cost [174]. Plant expression systems, represented by *N. benthamiana* and *N. tabacum*, on the other hand, require very minimal infrastructure and can be cultivated in the field or in greenhouses, hence lowering the cost of operations [175]. Besides the economic reasons above, plant-derived vaccines are highly scalable [176]. Plants being host species for vaccine candidates are grown at scale and in minimal consumption of resources; therefore, enabling an upscale rise in vaccine candidate production. Another very essential scalability factor would extend to the vaccine distribution part, in which cultivation can be performed locally to reduce transport costs and reduce dependency on cold-chain logistics, which happens to be one of the huge challenges in the traditional method of vaccine distribution [177]. Further, this also adds potential in edible vaccines as a factor of cost-effectiveness, in that vaccines are deliverable through food crops themselves, without needing complex delivery systems like injections.

Taken together, these advantages make plant-based vaccines an extremely cost-efficient and scalable alternative to supplement production for vaccines targeting both humans and animals.

### 5.2. Rapid Response to Pandemics and Emerging Diseases

One of the most attractive benefits of plant-based vaccines is their potential to act with great speed in the face of pandemics and newly emerging diseases. Compared to traditional vaccine production, which can take a very long time, sometimes months or even years, to increase production due to reliance on mammalian cell cultures or avian eggs, plant-based systems offer a much faster turnaround [178].

Indoor production of plant-based vaccines offers the possibility of producing vaccines in a reproducible, easily scalable manner. Plants for the production of such vaccines can be grown under strictly controlled conditions of light, temperature, and humidity in greenhouses or so-called plant factories, ensuring reproducibility of yields besides minimizing risks with regard to contaminants by pests and pathogens. This setup has the added advantage of being able to produce year-round, independent of seasonal or geographical constraints, and meets biosafety needs through containment of genetically modified plants. Indoor systems enhance quality control and give homogeneous vaccine output, hence making regulation easier to comply with, especially in rapid responses to global health challenges.

Such rapid manufacturing capacity is of particular importance when outbreaks occur because of new or re-emerging infectious diseases and require timely intervention in order to halt further spread. Plant systems allow for fast reprogramming in the event of new pathogens, since they can be genetically engineered to develop antigens to recently isolated viruses or bacteria [179].

### 5.3. Reduced Risk of Contamination with Human Pathogens

Perhaps most fundamentally, the development of vaccines within plants eliminates, almost by default, much risk of contamination from human pathogens [180]. Vaccine systems based on plant-based technologies offer a safer choice, since plants are naturally non-susceptible to the same human pathogens, therefore, significantly reducing the potential for contamination [181].

The use of plants to synthesize vaccines negates the need for materials like fetal bovine serum, derived from animals and typically used in more traditional cell culture systems. Such a system reduces the risks of zoonotic diseases (those which are transmitted between animals and humans) and satisfies other ethical concerns as it avoids using animals in the production process for biotechnology [105,182].

Also, plant-based systems are highly adaptable, enabling a rapid scale-up of production under controlled conditions. This ability to produce vaccines on a large scale while reducing the risk of contamination with human pathogens increases their suitability for global health programs [183].

### 5.4. Environmental Sustainability and Low Carbon Footprint

Plant-based vaccines offer major environmental sustainability and a low carbon footprint [184]. The manufacturing process of vaccines traditionally would involve highly energy-, water-, and raw material-intensive methods, such as mammalian cell cultures or chicken eggs, hence very resource-intensive and contributory to greenhouse gas emissions. Plant systems are environmentally sustainable as they require fewer resources, such as water and energy, and generate minimal waste compared to traditional biomanufacturing systems.

Plants used for vaccine production, like *N. benthamiana* and *N. tabacum*, can be grown in different environments, either in the field or in greenhouses, with very minimal chemical inputs. Unlike the conventional animal-based systems, plants do not require large-scale infrastructure; hence, they minimize the need for energy-intensive facilities. Moreover, plant-based vaccine production can be localized, reducing transportation and associated carbon emissions, especially in remote or underserved regions [185].

Moreover, plant-based vaccines contribute to sustainability by dispensing with animal-derived materials like fetal bovine serum, normally used in vaccine production. For example, making this change lowers the environmental footprint associated with cattle rearing or animal husbandry, thus, again providing more justification for adopting the plant-based technologies of vaccine manufacture as the way forward for ecological reasons [186].

In short, the ecological sustainability and lower carbon emissions related to plant-derived vaccines represent a significant advantage in concordance with international initiatives focused on reducing the environmental impacts of biotechnological production and increasing the number of more sustainable options in healthcare.

## 6. Challenges in Plant-Based Vaccine Development

### 6.1. Production Challenges

In addition to the issue of protein expression consistency, which is subject to variation depending on the plant host and/or growth conditions, low protein yields are one of the primary obstacles to the development of plant-based vaccines [187]. The specific challenges for transient expression plants are scalability and uniformity. Vaccine antigen production in plants would generally depend on factors such as plant species, growth environment, and specific methodologies of transformation utilized [188]. Such variability could, in turn, provide inconsistent yields of the target protein, hence not easy to standardize for the production and ensure the potency that vaccines normally would need. Unlike mammalian cell culture systems, where conditions can be tightly controlled, plant systems can be influenced by environmental factors such as temperature, light, and soil quality, all of which can affect protein expression levels [189,190].

In addition, it is a big challenge to produce high yields of the antigen of interest without metabolic imbalance specific to stably transformed plants [191]. Plants are complex organisms that have evolved for growth and reproduction, not for protein production; hence, directing a significant portion of their metabolic resources to produce foreign proteins can limit overall plant health and yield. Yield improvement methodologies, for example, the optimization of expression vectors or plant promoters that improve antigen expressions, have been developed though this is thought to yield in a high continuous matter [192].

The challenges regard the better methodology for the plant transformation, superior conditions of growing plants, more complete knowledge on primary metabolism of the plants for production assurances on higher and low-cost qualities.

### 6.2. Downstream Processing

Downstream processing remains one of the major challenges in plant-based vaccine development, mainly because of the complexity and cost of protein purification and standardization [193]. Following the production of recombinant proteins in plants, the extraction and purification process is considered critical to ensure the quality, safety, and efficacy of the final vaccine product [194]. Most plant-derived proteins are part of complex plant matrices, and the target antigen has to be purified from other plant proteins, carbohydrates, and lipids, which can be very labor-intensive and expensive [195].

One of the major problems is heterogeneity in plant-produced proteins. Unlike mammalian systems, plant expression systems can produce a variety of glycoforms that may affect the antigen’s function and immune response [196]. Achieving consistent glycosylation and folding of proteins in plant-based vaccines is challenging due to variability in plant growing circumstances and genetic backgrounds, which can result in batch-to-batch inconsistencies [197,198]. The standardization of plant-derived vaccines is challenging, necessitating comprehensive optimization of the purifying process and meticulous oversight of protein quality [199].

### 6.3. Regulatory Hurdles

Perhaps the most significant challenge in the development of plant-based vaccines is regulatory hurdles brought about by the present frameworks and their limitations to approve plant-derived biopharmaceuticals [200,201]. Since regulatory agencies, such as FDA and EMA, are set up based on conventional vaccines from animal or microbial systems, it becomes difficult for plant-based vaccines to meet some of the set standards in terms of safety and efficiency [202]. This novelty in the vaccine production sector using genetically engineered plants challenges the regulatory process because there are no standardized protocols that assess the safety of transgenic plants for environmental impact, allergenicity, and other unforeseen effects [203,204].

One of the key concerns is the approval of genetically modified organisms (GMOs), as many plant-based vaccines are produced using transgenic plants [205]. The regulatory authorities have strict policies for the release and consumption of GMOs, and every new product is put to rigorous testing to be declared safe for use. Most of the vaccines produced using plants take a long time before hitting the market due to a time-consuming and costly process of going through regulatory approval [206].

### 6.4. Public Acceptance

Public acceptance still is one of the most important challenges for the development and wide diffusion of plant-based vaccines, mainly because of ethical concerns and societal misconceptions about genetically modified plants [207]. Misconceptions at the social level about GMOs also play a role in skepticism towards vaccines made from plants. The public’s general view on GMOs is normally misguided by misinformation, fear of unnatural food sources, and an inability to draw a line between genetically modified food and biopharmaceutical products [208]. Public awareness of the safety, benefits, and ethical issues of plant-based vaccines is a prerequisite for these challenges to be met [209]. The transparent communication, education, and scientific outreach engaged in will dispel myths and foster a better-informed public discourse on the potential of plant-based vaccines to contribute to global health improvement.

## 7. Recent Innovations in Molecular Farming

Recent innovations in molecular farming are revolutionizing the plant-based vaccine development landscape and offering new avenues for the improvement of production systems [210]. Gene editing, nanoparticle-based delivery systems, and artificial intelligence represent some of the more significant recent advances that accelerate progress in the optimal development of plant expression systems and improvement of vaccine efficacy.

### 7.1. Advances in Gene Editing (e.g., CRISPR) for Optimizing Plant Systems

The development of gene editing tools, especially CRISPR-Cas9, has greatly improved this method by allowing for the precise introduction of changes into plant genomes [211]. This has led to advances in the reliability of plant recombinant protein production [212,213]. CRISPR technology allows the editing of plant DNA at very specific points, hence allowing optimization of particular traits relevant to protein production, such as yield, stability, and glycosylation profiles [214]. The application of CRISPR to plant genomes can achieve high and more consistent yields of vaccine antigens from plants—a problem that has long been haunting plant-based production platforms [215]. Additionally, this powerful gene editor is capable of ridding transgenic plants of unwanted characteristics, including allergenicity and immunogenicity, increasing safety and efficacy in plant-derived vaccines [216].

These exact modifications not only enhance the yield and quality but also the time period taken in order to genetically modify plants. This will include CRISPR application to optimize plant promoters that drive antigen expression and, thus, enable the production of proteins efficiently in plant tissues such as leaves, stems, and roots [217].

### 7.2. Development of Nanoparticle-Based Delivery Systems

**Figure 5:**
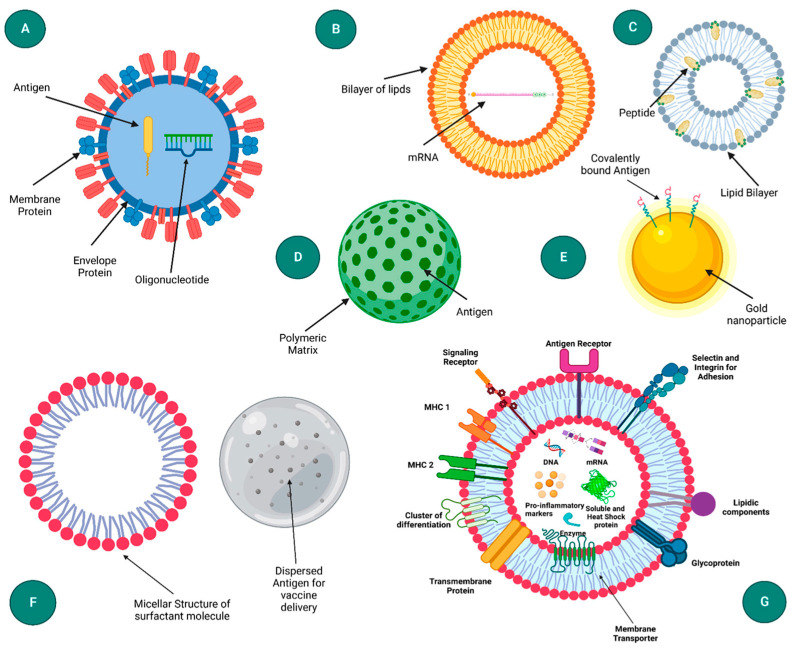
Schematic representation of different nanoparticle-based delivery systems: (**A**) VLP, (**B**) liposome, (**C**) Immune stimulating complexes (ISCOM), (**D**) polymeric nanoparticle, (**E**) inorganic nanoparticle, (**F**) emulsion, and (**G**) exosome. Copyright MDPI (2022) [218].

Nanoparticle-based delivery systems have emerged as a promising strategy for enhancing the efficacy of vaccines, including plant-based vaccines, by improving antigen stability, bioavailability, and targeted delivery. These nanoparticles, such as lipid, polymeric, and inorganic nanoparticles, protect vaccine antigens from degradation and enable controlled release for a sustained immune response [218] (Figure 5). In plant-based vaccines, nanoparticle carriers can facilitate oral or mucosal administration, making vaccination more efficient, non-invasive, and cost-effective.

Probably the most promising strategy is the utilization of non-infectious-derived VLPs from plant systems with a structure like a virus [219,220,221,222]. These can be engineered to display specific antigens and often serve as potent vaccines in vivo. This delivery method has been shown to improve the immune response by providing a more stable, targeted release of the vaccine into the body [29,223,224].

Other key approaches under consideration in the development of plant-based vaccines are nanoparticle-based delivery systems [225,226]. Most of the approaches use nanoparticles that encapsulate vaccine antigens, hence delivering them more efficiently and in a controlled manner inside the host cells [46,227,228]. Nanoparticles, such as lipid nanoparticles, gold nanoparticles, and chitosan nanoparticles, are combined with plant-derived proteins for enhanced bioavailability and stability of vaccines [229]. These nanoparticles can also protect the vaccine antigens from degradation to reach the target site in the body without deterioration [230]. Such systems may also enable edible vaccines whereby the antigen is encapsulated within a nanoparticle carrier in the plant tissue. This can allow for oral vaccine administration, thereby making vaccine administration non-invasive and less costly than conventional methods.

### 7.3. Integration of Artificial Intelligence in Plant Biotechnology

Artificial intelligence (AI) is a very recent and important tool for plant biotechnology, especially in optimizing plant-based vaccine production systems [231,232]. AI-powered algorithms, on the other hand, examine genomic data, protein structure, and conditions for growth to select an optimum combination that maximizes antigen expression by way of genetic modifications along with changes in environmental conditions [233]. Furthermore, using machine learning and deep learning methods, AI hastens the development process from design and testing to improvement in plant transformation systems [234]. The experimental data would also automatically analyze using such emerging technologies and consequently accelerate new vaccine development quite fast [235].

AI finds its way into high-throughput screening systems, where it predicts how different plant types will react to genetic modifications [236]. In this way, the best-performing plant lines can be selected quickly, reducing trial-and-error work. AI can also optimize growth conditions such as light intensity, temperature, and nutrient availability to make sure that the plants are at peak productivity during the production of vaccine antigens [237]. Besides accelerating research and development, AI can also point out the probable safety risks through the analysis of large datasets about biological interactions for the intended and unintended consequences of genetic modification [238]. This would help a great deal in ensuring better safety standards and hastening the regulatory process for approval in respect of plant-based vaccines.

Recent gene-editing innovations, nanoparticle-based delivery, and artificial intelligence have made immense improvements to the possibility of molecular farming in the manufacture of plant-based vaccines [239]. This would improve yield and consistency, with even safer production and more effective delivery.

## 8. Future Directions

### 8.1. Exploration of Personalized Vaccines Through Molecular Farming

Personalized medicine has been the catch-all phrase among all vaccine stakeholders, and plant-based vaccine technologies are unique in place to contribute to this emerging discipline [240]. Personalized vaccines take into consideration the genetic constitution, susceptibility to disease, and immune response profile at an individual level for vaccination strategies [241]. To sum up, molecular farming is the most viable platform to produce such products with a production capacity in the manufacture of recombinant protein and antigen production in plants. Using such a technique, the antigens can be genetically engineered to produce specific needs a patient may present. For instance, plants can produce anti-cancer vaccines that can attack tumor-specific antigens peculiar to a person’s cancerous cells [242]. Being an approach that would give an opportunity to rapidly produce customized vaccine candidates, it caters to the precision required in treatments such as cancer vaccines. Indeed, a recent study also investigated plant-based vaccines for oral delivery of type 1 diabetes-related autoantigens by evaluating oral tolerance mechanisms and disease prevention in a non-obese diabetic (NOD) mouse model [243]. Furthermore, plant-based platforms offer a cost-effective and scalable manufacturing method for these tailored vaccines in combination with more recent advanced technologies involving CRISPR gene editing and accompanying bioinformatic tools to analyze genetic data. This could significantly lower the cost of production and make personalized immunotherapy a globally applicable approach.

### 8.2. Synergistic Approaches: Combining Plant-Based Vaccines with Nanotechnology and Other Platforms

With the help of nanoparticles, plant-based vaccines can also serve as carriers to enhance the immunogenicity of antigens derived from plants [216]. For example, nanoparticle formulation can envelope plant-produced VLPs for better mimicry of the pathogen structure, thus, inducing an improved immune response [244,245]. Nanoparticles in vaccine formulation enhance vaccine stability, protect antigens from environmental degradation, and increase shelf-life without refrigeration.

Further synergy may be obtained by combining plant-based vaccines with other platforms, such as mRNA vaccines. For example, plant systems could serve in the biomanufacturing of mRNA vaccines by synthesizing viral proteins in plants, which afterward can be utilized for the production of mRNA, already embedded in the vaccine formulations [246].

### 8.3. Expanding Applications Beyond Human Vaccines

Most plant vaccines are under development therapeutics and diagnostic tools are on the rise. Plant platforms are also tested for their possible use in therapeutic vaccines, especially against cancer and other chronic diseases. These vaccines trigger the immune response to attack cancer cells or the agent causing a disease. Chronic diseases such as HIV/AIDS and autoimmune diseases could also benefit from plant-based immunotherapies, where the plant platform can be used to deliver specific immune-modulating agents [156,247]. Furthermore, plant-based systems are being studied as platforms for diagnostics, e.g., plants can be designed to produce antibody-like proteins for diagnosis of infectious diseases, serving as a diagnostic tool far simpler and cheaper than the diagnostics themselves [248,249].

### 8.4. Addressing Global Health Inequities and Pandemic Preparedness

Plant vaccines hold great promise, especially in attempting to bridge gaps in health inequities on a global scale and preparing better against pandemic outbreaks [250,251]. Scalability in the production of plant-based vaccine systems also ensures rapid deployment even in the low-resource environment where it would be far more challenging for the scaling-up process using the conventional vaccine production platforms [252]. Royal et al. investigated a SARS-CoV-2 vaccine candidate using plant-based manufacturing and tobacco mosaic virus-like nanoparticles [253]. This study illustrates that by combining the SARS-CoV-2 receptor-binding domain with a tobacco mosaic virus nanoparticle, the CoV-RBD121-NP vaccine has high ACE2 binding, neutralizing antibody recognition, and 12-month stability. This finding suggest a stable and scalable COVID-19 vaccination.

## 9. Conclusions

Plant-based vaccines have the potential to revolutionize the face of immunization by offering an affordable, scalable, and ecologically friendly alternative to traditional methods of vaccine production. The vaccines can be produced in a very short time, hence reducing the time taken to respond to emerging infectious diseases and pandemics, as was realized during the rapid development of Medicago’s Covifenz for COVID-19. The capacity for vaccine production in plants has great potential to level the playing field with regard to global health disparities by providing affordable immunization options, especially in resource-poor settings where traditional vaccine production infrastructure may not exist. Since then, plant-based systems have expanded well beyond human vaccines to include applications in veterinary medicine, cancer immunotherapy, and diagnostic methods. Molecular farming for personalized vaccines, added to other developments such as nanotechnology and artificial intelligence, will further expand the boundaries of what has been believed possible in immunization strategies to make vaccines even more personalized, efficient, and accessible. The complete exploitation of plant-based vaccines does, however, require the overcoming of some restrictions present nowadays. Those inhibit its application, including low yields of protein, challenges in downstream processing, and regulatory problems related to the use of genetically modified organisms. Some of those hurdles have been conquered with continued innovation in plant biotechnology hand in hand with research institutions, regulatory organizations, and the pharmaceutical industry. With these obstacles finally overcome, plant-based vaccines have provided groundbreaking responses for global health in a sustainable and scalable platform that could meet the world’s needs in the battle against infectious diseases.

## Figures and Tables

**Table 1:** Plant-Based Vaccines for Infectious Diseases.

Target Disease	Antigen	Plant Host	Vaccine Type	Animal Species for Study	Reference
COVID-19	SARS-CoV-2 Spike Protein	*N. benthamiana*	VLP	Mice	[132]
COVID-19	SARS-CoV-2 Spike Protein	*N. benthamiana*	VLP	Golden Syrian hamsters	[133]
Influenza	Hemagglutinin (HA)	*N. benthamiana*	VLP	Healthy Subjects	[134]
Malaria	Pfs25	*N. benthamiana*	VLP	Mice	[131]
Malaria	Pfs25	*N. benthamiana*	VLP	Healthy Subjects	[135]
Norovirus	Narita 104 virus capsid protein	*N. benthamiana*	VLP	Mice	[136]
Hepatitis B	HBsAg	*Lactuca sativa*	Subunit	Mice and Healthy Subjects	[58]
Rabies	Rabies virus glycoprotein (G protein)	*N. benthamiana*	Subunit	Mice	[137]
Cholera	Cholera Toxin B (CTB)	*N. benthamiana*	Subunit	Mice	[138]

**Table 2:** Examples of Plant-Based Vaccines in Veterinary Medicine.

Target Disease	Animal Species	Plant Host	Antigen	Notes	Reference
Newcastle Disease	Poultry	Tobacco	Hemagglutinin-Neuraminidase (HN) protein	Provides immune protection in chickens	[166]
Foot-and-Mouth Disease	Livestock	Alfalfa	VP1 capsid protein	Effective immunogenicity in cattle and pigs	[167]
Rabies	Dogs	Tomato, tobacco	Rabies glycoprotein	Offers a cost-effective alternative for rabies prevention	[168]
Avian Influenza	Poultry	Duckweed, tobacco	Hemagglutinin (HA) protein	Prevents viral outbreaks in poultry populations	[169]

## Data Availability

Not applicable.
